# Linking *Bacillus cereus* Genotypes and Carbohydrate Utilization Capacity

**DOI:** 10.1371/journal.pone.0156796

**Published:** 2016-06-07

**Authors:** Alicja K. Warda, Roland J. Siezen, Jos Boekhorst, Marjon H. J. Wells-Bennik, Anne de Jong, Oscar P. Kuipers, Masja N. Nierop Groot, Tjakko Abee

**Affiliations:** 1 TI Food and Nutrition, Wageningen, The Netherlands; 2 Laboratory of Food Microbiology, Wageningen University, Wageningen, The Netherlands; 3 Wageningen UR Food & Biobased Research, Wageningen, The Netherlands; 4 Center for Molecular and Biomolecular Informatics, RadboudUMC, Nijmegen, The Netherlands; 5 Microbial Bioinformatics, Ede, The Netherlands; 6 NIZO Food Research B.V., Ede, The Netherlands; 7 Department of Molecular Genetics, University of Groningen, Groningen, The Netherlands; Academia Sinica, TAIWAN

## Abstract

We characterised carbohydrate utilisation of 20 newly sequenced *Bacillus cereus* strains isolated from food products and food processing environments and two laboratory strains, *B*. *cereus* ATCC 10987 and *B*. *cereus* ATCC 14579. Subsequently, genome sequences of these strains were analysed together with 11 additional *B*. *cereus* reference genomes to provide an overview of the different types of carbohydrate transporters and utilization systems found in *B*. *cereus* strains. The combined application of API tests, defined growth media experiments and comparative genomics enabled us to link the carbohydrate utilisation capacity of 22 *B*. *cereus* strains with their genome content and in some cases to the *panC* phylogenetic grouping. A core set of carbohydrates including glucose, fructose, maltose, trehalose, N-acetyl-glucosamine, and ribose could be used by all strains, whereas utilisation of other carbohydrates like xylose, galactose, and lactose, and typical host-derived carbohydrates such as fucose, mannose, N-acetyl-galactosamine and inositol is limited to a subset of strains. Finally, the roles of selected carbohydrate transporters and utilisation systems in specific niches such as soil, foods and the human host are discussed.

## Introduction

Metabolic activity and growth of bacteria requires energy that can be acquired during carbohydrate catabolism. Carbohydrates can be present in nature in complex forms such as polysaccharides including starch, cellulose or glycogen, but also as mono- and disaccharides exemplified by glucose and sucrose. Efficient use of available carbohydrates by microorganisms is determined by the different types of carbohydrate transporters and utilization systems encoded by chromosome- and plasmid-located genes and gene clusters [1,2]. In addition, expression and regulation of sugar utilisation systems can be affected by environmental conditions such as oxygen availability and temperature, and carbon catabolite repression (CCR) [1,3,4].

Systems for carbohydrate uptake and metabolism predicted based on genome analysis can vary significantly between species and strains as shown for *Escherichia coli* [5], *Bacillus subtilis* [6], *Shewanella* spp. [7] and lactic acid bacteria including *Lactobacillus plantarum* [2,8], *Lactobacillus casei* [9], *Oenococcus oeni* [10] and *Lactococcus lactis* [11]. In gram-positive bacteria, these genes are generally organized in functional gene cassettes or modules, encoding a single or multi-component transporter (1–4 subunits), enzymes for sugar breakdown and a transcriptional regulator, with each cassette (semi)-specific for a certain carbohydrate [2,12,13]. A cassette is a functional unit, and can consist of more than one operon. The presence or absence of entire cassettes can be highly variable, and is thought to reflect adaptation to growth on particular sugar substrates in specific niches such as soil and plant rhizosphere, foods and food processing environments, and the human host [1,13,14]. Often carbohydrate metabolism cassettes are clustered together on the chromosome, in so-called lifestyle or sugar islands associated with colonisation of specific niches such as dairy environments [8,11,13,15]. The deviating base composition of these genomic islands and cassettes suggests their acquisition via horizontal gene transfer [16]. The increasing availability of microbial genome sequences has stimulated comparative analysis of carbohydrate utilisation capacity, on the one hand to optimize performance of fermentation starter bacteria and probiotics, and on the other hand, to understand pathogen behaviour in the environment and inside the host, enabling development of better preservation and control measurements in food processing and clinical settings [12,14,17].

Three main types of carbohydrate transporters can be found in bacteria: ATP-binding cassette (ABC) transporters, permeases and phosphoenol-pyruvate (PEP)-dependent carbohydrate phosphotransferase systems (PTS) that catalyse the transport and phosphorylation of numerous monosaccharides, disaccharides, amino sugars, polyols, and other sugar derivatives. To carry out its catalytic function in sugar transport and phosphorylation, the PTS uses PEP as an energy source and phosphoryl donor. The phosphoryl group of PEP is usually transferred via four distinct proteins (domains) to the transported sugar bound to the respective membrane component(s) (EIIC and EIID) of the PTS [4]. Notably, the PTS have also numerous regulatory roles for example in carbon and nitrogen metabolism, antibiotic resistance, biofilm formation, toxin production and virulence [4,18,19]. Recently sugar acids were shown also to be transported by tripartite ATP-independent periplasmic transporters (TRAPs) [20].

Utilisation of a broad range of carbon and energy resources, either commonly present in the environment or associated with specific niches, can support not only ubiquitous presence but also facilitate transmission and pathogenicity of food-borne pathogens such as *Bacillus cereus* [21]. The ability to thrive in different environments such as soil, food and food processing environments and human gastrointestinal tract is supported by a range of carbohydrate transport and utilisation systems that indirectly facilitate transmission from these environments to the human gastrointestinal tract.

*B*. *cereus* is a representative of the *Bacillus cereus sensu lato* group comprising seven closely related species of spore formers that are associated with different environments and contain both non-pathogenic and pathogenic bacteria including *Bacillus mycoides*, *Bacillus pseudomycoides*, *Bacillus weihenstephanensis*, *Bacillus thuringiensis*, *Bacillus anthracis*, *B*. *cereus*, and the recently identified *Bacillus cytotoxicus* [22,23]. *B*. *cereus sensu lato* group members have been classified into one of seven phylogenetic groups introduced by Guinebrettiere et al. [22], according to which *B*. *cereus* strains can be found in five of these groups, i.e., group II, III, IV, V and VI, covering a broad range of growth temperatures from 5 to 50°C.

*B*. *cereus* is often associated with spices and foods such as rice, cereals and dairy products [21]. Transmission of *B*. *cereus* is facilitated by the production of highly stress resistant dormant spores that are triggered to germinate in nutrient-rich conditions via an interplay of (combinations of) germinants and a variety of so-called nutrient germinant receptors [24]. Germination and outgrowth of *B*. *cereus* spores into vegetative cells in foods may lead to food spoilage and safety issues [25,26]. The vegetative cells of *B*. *cereus* produce toxins in food before the ingestion or in the small intestine leading usually to mild and self-limiting symptoms but in rare instances it can lead to life-threatening situations [27]. *B*. *cereus* clinical isolates have been associated with gastrointestinal infections and non-gastrointestinal infections particularly in immune compromised patients or neonates resulting in wound infections, ocular infections and bacteraemia [21,25–27]. Genome analysis of selected model strains pointed to *B*. *cereus* specialisation in protein metabolism, suggesting that it has adapted towards a symbiotic or parasitic life cycle [25,28–30]. *B*. *cereus* strains carry genes for utilisation of mono- and disaccharides such as glucose and trehalose [28,31], and polysaccharides such as starch [31]. Moreover performance in selected niches has been associated with transport and metabolism of specific carbohydrates such as glucose-6-phosphate [32].

Despite the putative role of selected carbohydrates in growth and survival in a range of environments, a detailed comparative genotypic and phenotypic analysis that includes multiple genome-sequenced *B*. *cereus* strains representing the different phylogenetic groups described by Guinebrettiere et al. [22] has not been reported up to now.

The current study provides an overview of the different types of carbohydrate transporters and utilization systems in 20 newly sequenced *B*. *cereus* food isolates and model *B*. *cereus* strains ATCC 14579 and ATCC 10987, and links these to metabolic capacity using API tests and/or defined media with selected carbohydrates as carbon and energy sources. Additionally, roles of selected carbohydrate transporters and utilisation systems in specific niches are discussed.

## Materials and Methods

### Strains used

Twenty *B*. *cereus* strains previously isolated from food products and food processing environment (NIZO Culture Collection, Ede, the Netherlands) and two reference strains *B*. *cereus* ATCC 14579 and *B*. *cereus* ATCC 10987 obtained from the American Type Culture Collection (ATCC) (S1 Table) [33,34] were streaked from -80°C stock on Bacto Brain Heart Infusion (BHI; Beckton Dickinson, Le Point de Claix, France) agar plates and incubated at 30°C overnight to obtain single colonies. Based on Average Nucleotide Identity (ANI typing; [35]) criteria strain B4117 (LJKG00000000.1), was recently re-classified by NCBI as *Bacillus mycoides*, phenotype information should be included to confirm this.

### API growth tests

API 50CH (BioMerieux, France) test was used in combination with API 50CHB/E medium (BioMerieux, France) according to manufacturer’s instruction. Shortly, single colonies were used to inoculate API 50CHB/E medium to a turbidity equivalent of 2 McFarland. Test strips were filled with inoculated medium, incubated at 30°C, and checked for media colour change after 24 and 48 h. For each strain, three independent repetitions were performed.

### Defined media growth experiments

Ten ml of BHI was inoculated with a single colony and incubated overnight at 30°C with aeration at 200 rpm. One ml of overnight grown culture was pelleted in a table top centrifuge (4000 rpm, 1 min), washed with Peptone Physiological Salt buffer (PFZ; Tritium Microbiologie, the Netherlands) and resuspended in 1 ml of PFZ. 96-well plates were filled with 20 μl of 10x diluted washed culture and 180 μl of test media. Plates were then transferred into a plate reader (Tecan Infinite F200 Pro, Austria) for incubation at approximately 200 rpm at 30°C. OD_595_ was measured every 10 min and read outs were used to score for positive or negative growth. Chemically defined Y1 medium [36] was slightly modified by omission of lactic acid and glucose, and lowering glutamate concentration down to 1 mM. The modified medium was supplemented with one of the following carbon sources (final concentrations in mM): glucose (12.5 mM), sodium gluconate (25 mM), glycerol (50 mM), L-fucose (25 mM), glucose-6-phosphate (25 mM), myo-D-inositol (25 mM), and N-acetyl-D-galactosamine (18 mM). Non-supplemented modified medium was used as control. Three technical replicates were performed for each condition.

### Comparative genomics

In total, the genomes of 33 *B*. *cereus* strains were included in the comparative analysis. Next to the 20 newly sequenced food-spoilage *B*. *cereus* strains [33,34] used in the growth experiments, also 13 *B*. *cereus* genomes including *B*. *cereus* ATCC 14579 and *B*. *cereus* ATCC 10987 were obtained from the NCBI-Genbank database (http://www.ncbi.nlm.nih.gov/genome/genomes/157) and compared to each other and to the genome of reference strain *Bacillus subtilis* 168 (S1 Table). All genomes were (re)annotated using RAST [37] to allow a better comparison of all annotations.

Orthologous groups (OGs; i.e. gene families) in the genomes were determined using OrthoMCL [38]. A database (in MS Excel) was built containing aligned information about the location and length of orthologous genes and proteins (i.e. on which contig and base pair position on the assembled genome it is present) of the newly sequenced and published reference genomes. Moreover, for every OG a multiple sequence alignment was made on te amino acid level using Muscle [39] to facilitate identification of pseudogenes (encoding incomplete proteins).

The carbohydrate utilization systems of *B*. *subtilis* 168 listed in the Subtiwiki database (highly curated) [40] were initially used to search for orthologous systems in the *B*. *cereus* OG table. Additional genes and systems were found with keyword searches and genome context analysis. Subsequently, selected genes/proteins were manually curated by comparison against sequence databases (using e.g. NCBI-BLASTP; http://blast.ncbi.nlm.nih.gov/), family/domain databases (using e.g. InterPro [41]), enzyme databases (using e.g. Brenda [42]) and pathway databases (using e.g. KEGG [43]). Gene cassettes and gene synteny were visualized using MGcV (Microbial Genome context Viewer) [44]. Details of the carbohydrate utilization cassettes in the OG database can be found in S2 and S3 Tables.

### Phylogeny

For the construction of a phylogenetic tree, next to the genomes of 33 *B*. *cereus* strains and *B*. *subtilis* 168, additional circular reference genomes of 18 strains from the *B*. *cereus sensu lato* group were included, i.e. 6 *B*. *anthracis* strains, 11 *B*. *thuringiensis* strains and 1 *B*. *weinenstephanensis* strain. On the basis of an OrthoMCL analysis of all these genomes, all OGs were selected with a single orthologous gene in each of the different genomes. The protein sequences of these core OGs were aligned using Muscle [39] and alignment positions with amino acid differences were selected and stored in a single artificial protein sequence. This protein sequence was used as a basis to generate a whole-genome MLST phylogenetic tree using FastTree [45].

Affiliation of 51 *B*. *cereus sensu lato* group strains to the phylogenetic groups (I to VII) proposed by Guinebretière et al. [22] was performed by extraction of *panC* gene sequences from the OG group and implementing in the online tool (https://www.tools.symprevius.org/Bcereus/english.php).

## Results

### Genome statistics and phylogeny

**Fig 1:**
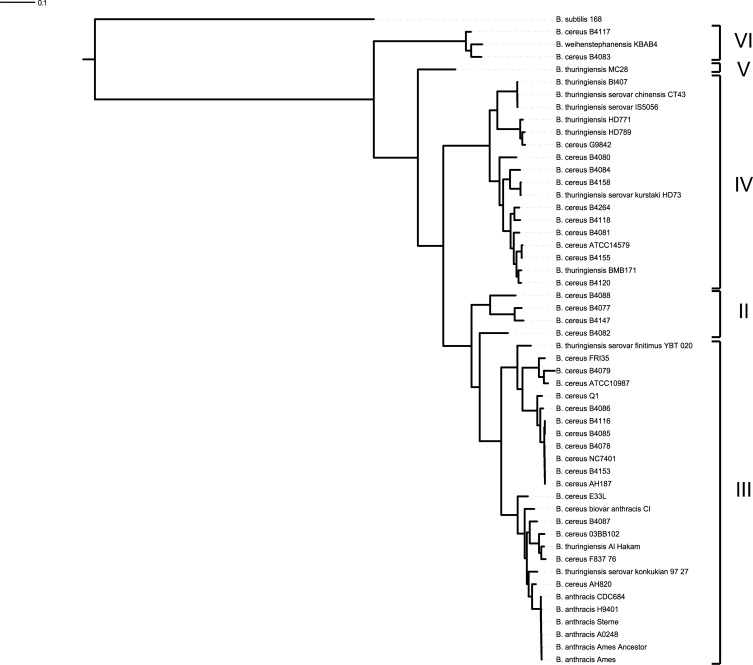
A phylogenetic tree based on the core genome of 20 newly sequenced *B*. *cereus* strains used in this study and 31 previously described strains of the *B*. *cereus sensu lato* group used for reference purposes; *B*. *subtilis 168* was used as outgroup. Roman numbers indicate phylogenetic groups defined previously by Guinebretière [22].

The 33 *B*. *cereus* genomes were found to contain 1775 core ortholog groups (OGs), i.e., orthologous genes that occur in all genomes only in one copy. A phylogenetic tree was built based on differences in all encoded core proteins derived from 33 *B*. *cereus* genomes and an additional 18 reference genomes from the *B*. *cereus sensu lato* group and *B*. *subtilis* 168 (Fig 1). The phylogenetic clustering of the strains is in line with the phylogenetic division of the *B*. *cereus sensu lato* group members based on the *panC* polymorphism published by Guinebretière [22] and the *B*. *cereus* strains of our study were found to belong to groups II, III, IV and VI.

### Growth experiments

**Fig 2:**
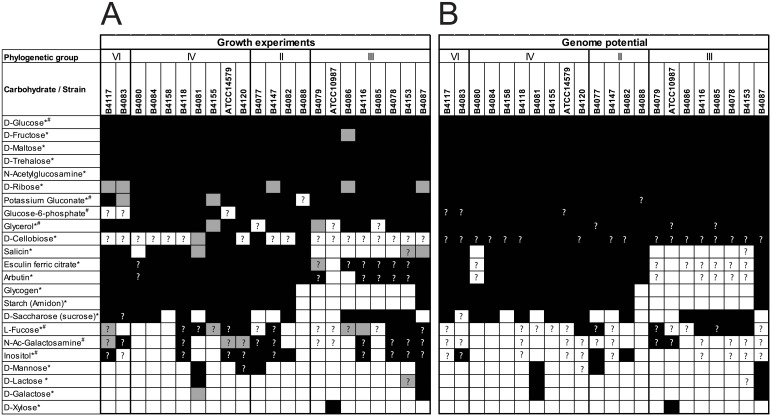
*B*. *cereus* carbohydrate utilisation based on growth experiments and genome potential. (A) *B*. *cereus* growth on different carbohydrates as determined by an API 50CHB/E test (*) and/or in defined media (#). Black: positive growth in at least two out of three replicate reactions; grey: positive growth in one out of three replicate reactions or doubtful reaction; white; no growth. (B) *B*. *cereus* genome potential. Black: at least one cassette present; white, no cassettes identified. Question marks indicate discrepancies between predicted and experimentally observed phenotypes. Footnote: No colour change (hence no growth) observed for all strains in API 50CHB/E on amygdalin, D-adonitol, arabinose, arabitol, D-fucose, D-lyxose, D-mannitol, D-melezitose, D-melibiose, D-raffinose, D-sorbitol, D-tagatose, D-turanose, dulcitol, erythritol, gentiobiose, gluconate, glycerol, inositol, inulin, L-fucose, L-rhamnose, L-sorbose, L-xylose, methyl-α-D-glucopyranoside, methyl-α-D-mannopyranoside, methyl-β-D-xylopyranoside, potassium 2-ketogluconate, potassium 5-ketogluconate, and xylitol.

In the API assay, all 22 tested *B*. *cereus* strains were found to grow on D-glucose, D-fructose, D-maltose, D-trehalose, N-acetylglucosamine and D-ribose, with the majority of the strains growing on sucrose, arbutin, esculin, salicin, and starch/glycogen. Only some of the strains grew on other carbohydrates, such as, lactose, mannose, galactose, cellobiose, and only *B*. *cereus* ATCC 10987 grew on xylose (Fig 2A). Growth studies in aerated defined media showed additional growth on fucose, glycerol, inositol, and gluconate for subsets of strains.

### Genome mining

**Fig 3:**
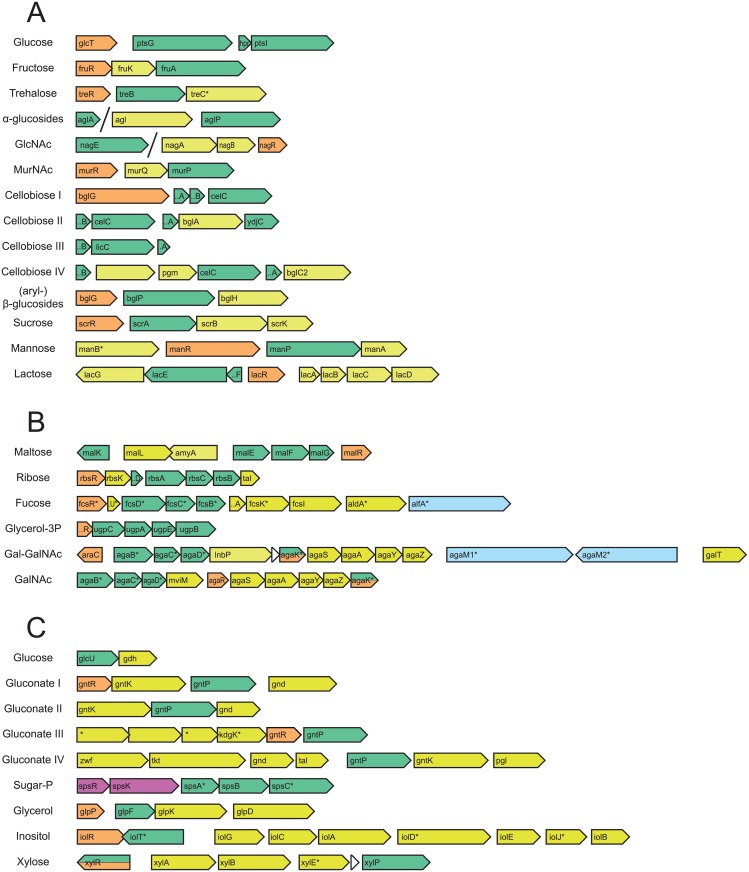
Predicted carbohydrate utilization gene cassettes. (A) PTS transporters. (B) ABC transporters. (C) secondary transporters (permeases). Colour coding for encoded proteins: green, transporters; yellow, intracellular enzymes; light blue, extracellular enzymes; orange, regulators; purple, two-component regulator. * genes with newly predicted function.

The *B*. *cereus* genomes were explored for presence of genes and gene cassettes encoding enzymes required for growth on the tested carbohydrates. The carbohydrate utilization systems of *B*. *subtilis* 168 listed in the Subtiwiki database (highly curated) were used to search for ortholog systems in the *B*. *cereus* OG table, and additional cassettes were identified by keyword searches and manual annotation. The identified cassettes are shown schematically in Fig 3, and the encoded functions are summarized in S2 Table. In general, very few pseudogenes were identified, and hence nearly all carbohydrate utilisation systems are predicted to be functional (details in S3 Table). Most of the pseudogenes were found in the *B*. *cereus* ATCC 14579 genome, but since the corresponding genes are functional based on the experimental data, the non-interrupted fragments of the pseudogenes may still be functional. The various *B*. *cereus* carbohydrate utilization systems are described below.

#### Carbohydrates commonly utilised by B. cereus

All of the 22 tested *B*. *cereus* strains grow on glucose (Fig 2) corresponding to the presence of a gene cassette encoding a glucose-specific PTS and a BglG family transcriptional anti-terminator (Fig 3A, **Table A in** S2 Table). Moreover, all *B*. *cereus* genomes harbour a cassette encoding a glucose permease GlcU and a glucose 1-dehydrogenase Gdh (Fig 3C, **Table C in** S2 Table). In addition, a permease annotated as glucose/mannose:H+ symporter (OG_5022) is present in 5 of the experimentally tested strains and reference strains 03BB102, AH820, and F837_76 (S3 Table).

All *B*. *cereus* strains carry four gene cassettes, each encoding a PTS transporter, an enzyme for sugar catabolism, and a transcriptional repressor, specific for transport and utilisation of fructose (*fruRKA*), trehalose (*trePAR*), N-acetyl-glucosamine (*nagABR*) and N-acetyl-muramic acid (MurNAc; *murPQR*) (Fig 3A, S2 and S3 Tables). Presence of those gene cassettes corresponds with the ability to utilise fructose, trehalose, and N-acetyl-glucosamine by all experimentally validated strains (Fig 2).

Additionally, all tested *B*. *cereus* strains grow on maltose, a disaccharide (Fig 2). The two gene cassettes for utilization of maltose present in *B*. *subtilis* genomes, *malARP* and *yvdEFGHIJKLM*, are absent in the *B*. *cereus* genomes. However, there is an equivalent, but different maltose/maltodextrin utilization cassette in all selected *B*. *cereus* genomes, encoding a maltose/maltodextrin ABC transporter MalEFGK, two enzymes for degradation of maltose polymers (i.e. an alpha-glucosidase/oligo-1,6-glucosidase, and a neopullulanase/alpha-amylase), and a maltose operon transcriptional regulator MalR (Fig 3B, **Table B in** S2 and S3 Tables).

Finally, a complete and intact ribose utilization gene cassette *rbsRKDACB*, encoding an ABC transporter (RbsDACB), a ribokinase RbsK and a ribose operon transcriptional regulator RbsR is present in all *B*. *cereus* as well as in all considered *B*. *cereus sensu lato* group genomes and *B*. *subtilis* 168. All *B*. *cereus* strains grow on ribose (Fig 2), with API tests of five strains being not conclusive. The latter observation may be linked to the in general poor readability of the ribose reaction in the API test.

#### Soil/plant related carbohydrates

Cellobiose and xylose can function as energy sources and are common components of plant cell walls that due to the plant decay process can be found in soil, while sucrose is a naturally occurring carbohydrate found in many plants.

*B*. *cereus* genomes encode four gene cassettes annotated as cellobiose-specific PTS (Fig 3A, **Table A in** S2 and S3 Tables) with three of these found in all selected *B*. *cereus* strains. The fourth putative cellobiose-specific cassette is present only in strains B4082 and AH820, and consists of a PTS transporter, a 6-phospho-beta-glucosidase and a phosphoglycerate mutase. The conserved presence of the first three PTS cassettes in all *B*. *cereus* genomes does not correlate with growth in the API test, as only 5 *B*. *cereus* strains were positive for growth on cellobiose (Fig 2). This could be explained by incorrect annotation of these PTS. For example, alternative annotations given in NCBI data base are diacetylchitobiose-specific (= glucosamine units) for the first gene cluster, and lichenan-specific (= complex branched glucose polymer) PTS for the second and third gene clusters. The beta-glucoside PTS (*bglP*, OG_5777) is another candidate for the import of cellobiose (a beta-glucoside), but the *bglP* gene was found only in *B*. *cereus* strains B4077, B4083, B4088, B4117, E33L and FRI_35, which also does not agree with the API growth results on cellobiose. Thus further work is required to elucidate the roles of these clusters in carbohydrate transport and metabolism.

Notably, the previously identified gene cassette for xylose uptake and utilization [31] was found only in ATCC 10987 (and few other *B*. *cereus* strains (i.e. FT9, F3162-04) not included in our study) and this was indeed the only strain growing on D-xylose (Fig 2). The corresponding cassette encodes a xylose permease XylP, a xylose isomerase XylA, a xylulose kinase XylB, a xylose 1-epimerase XylE and a ROK family transcription regulator XylR (Fig 3C, **Table C in** S2 and S3 Tables). The origin of the cassette is not known, but it replaces gene cassettes for nitrate reductase and molybdenum cofactor biosynthesis [28] which are present in that genomic region in all other *B*. *cereus* genomes studied here, except strains B4079, B4081 and B4147.

Fourteen *B*. *cereus* strains were found to grow on sucrose, whereas 8 strains did not grow (Fig 2). Mols et al. [31] suggested that growth of *B*. *cereus* ATCC 14579 on sucrose was linked to a gene cassette composed of a sucrose-specific PTS named SacP, a sucrose-6-phosphate hydrolase SacA, a fructokinase FruC, and a sucrose operon repressor SacR (BC0773-BC0776). The corresponding 4 genes are also present in *B*. *subtilis* 168 (but not in a single cassette) and are known to be responsible for growth on sucrose [46]. Indeed an almost perfect correlation was found between the presence of this sucrose utilization cassette in *B*. *cereus* strains and their ability to grow on sucrose (13 of 14 strains) (Fig 3A, **Table A in** S2 Table). Half of the publicly available *B*. *cereus* genomes also contain this cassette, suggesting that growth on sucrose is not a rare trait in *B*. *cereus* (S3 Table).

#### Food related carbohydrates

Bacteria present in soil are commonly transmitted via raw materials of plant (vegetable) or animal (milk, egg, meat) origin into food products. In general, raw materials and final food products are considered nutritious commodities. However, some groups of products may offer limited carbohydrate supplies, for example staple foods contain mainly starch while dairy products contain mainly lactose.

Starch is a polymer of glucose units, and consists of the linear-chain amylose and the branched-chain amylopectin. An analogue of starch, glycogen, has a similar structure to amylopectin, but more extensively branched and compact than starch. There is a glycogen biosynthesis gene cassette *glgPADCB* present in all *B*. *cereus* genomes, however 8 of the *B*. *cereus* strains are not able to utilise glycogen and starch (Fig 2). The genome of *B*. *cereus* ATCC 14579 contains an alpha-amylase gene (*amyS*, BC3482, COG0366) which was proposed to support growth on starch [31]. Alpha-amylase (EC 3.2.1.1) breaks down polysaccharides, ultimately yielding maltotriose and maltose from amylose, or maltose and glucose from amylopectin. The presence of the *amyS* gene (OG_4318) in the *B*. *cereus* strains correlates exactly with growth on starch or glycogen, indicating that this is the sole gene responsible for degradation of these polymeric substrates (Fig 2B, S3 Table). Moreover, this gene is absent in the *B*. *cereus* genomes available in the public database (strains E33L, FRI_35, NC7401, AH187 and Q1), suggesting that these strains will also not grow on starch/glycogen. The encoded protein AmyS of 513 residues is most likely secreted, since it has a signal peptide and a typical signal peptidase cleavage site (AYA|D). The extracellular degradation of starch or glycogen will lead to release of glucose units which can be accumulated by the maltose and glucose uptake systems and subsequently metabolised as described above.

*B*. *cereus* is commonly associated with dairy environments, and therefore we tested for utilisation of lactose, the main carbohydrate present in milk [47]. From our set of isolates, strains B4081 (isolated from Provolone sauce) and B4087 (isolated from pea soup) consistently grow on lactose (Fig 2), corresponding exactly with the presence of a lactose utilization gene cassette *lacGEFRABCD* in their genomes (Fig 3A). This cassette encodes a lactose PTS (LacEF), a 6-phospho-beta-galactosidase LacG, a lactose operon repressor LacR, a galactose-6P isomerase LacAB, a glucokinase and two enzymes of the tagatose pathway, i.e. tagatose-6-phosphate kinase LacC and tagatose 1,6-bisphosphate aldolase LacD. In both cases, the lactose utilization genes are predicted to be located on a 26–28 kb plasmid, and all encoded proteins are highly similar, suggesting that these 2 strains contain the same lactose plasmid. The 9-kb lactose utilization cassette is located on a 16-kb transposon which is flanked by Tn1546 transposases and resolvases. Parts of this plasmid, but not the lactose cassette, are also present in genomes of other *B*. *cereus* strains of this study (data not shown). A BLASTP analysis to the NCBI database shows presence and 100% protein sequence identity of this lactose cassette in *B*. *cereus* strains m1293, VD102, VD140, NVH 0075–95, and MHI 86 (S4 Table). The next matches to these proteins are found in *Carnobacterium* and various Bacilli, with 55–75% amino acid sequence identity, while the corresponding proteins on *Lactococcus lactis* plasmids have only 45–65% identity.

Notably, only strains B4087 and B4081, though poorly, grow on galactose (Fig 2). We could not find any genes or gene cassettes annotated as specific for uptake of galactose in any *B*. *cereus* genome. Since these are the only two strains that also grow on lactose, they may also be able to utilize free galactose via the lactose PTS, as lactose PTS was previously shown to transport galactose, albeit less efficiently in lactococci [48] and streptococci [49,50].

#### Host related carbohydrates

Food-borne human pathogens invading the intestinal tract and/or host cells depend on the availability of simple carbohydrates and/or degradation of complex host-associated carbohydrates/polymers, present for example in mucus. Typical host-derived carbohydrates may include fucose, mannose, N-acetyl-galactosamine and inositol [51–53].

L-fucose is a sugar present in human milk as building block of oligosaccharides, mucins and other glycoconjugates in the intestinal epithelium [54]. None of the strains grew on D-fucose or L-fucose in the API test, but in the defined medium growth experiments with aeration 8 strains showed growth on L-fucose, while 4 strains showed questionable growth (Fig 2). Fucose transport via a permease, an ABC transporter or a PTS transporter and subsequent utilization have been described in Gram-negative [55–57] and Gram-positive bacteria [54,58]. Additionally, extracellular alpha-fucosidases have been characterized from several bacilli [59–61], including *B*. *cereus* [62]. We identified a cassette comprising 10 genes for fucose utilization (Fig 3B, S3 Table), that includes a fucose ABC transporter FcsBCD, in seven of experimentally verified *B*. *cereus* strains and in genomes of Q1, NC7401, and AH187. This cassette encodes an extracellular alpha-L-fucosidase AlfA (**Table B in** S2 Table), suggesting that fucose can be cleaved from complex oligosaccharides, and then taken up into the cell and metabolized, ultimately to dihydroxyacetone phosphate and lactate, since a lactaldehyde dehydrogenase AldA is also encoded, next to an L-fucose isomerase FcsI, an L-fuculose kinase FcsK, an L-fuculose-6P aldolase FcsA, and a LacI family transcription regulator FcsR. Not all strains caring the complete cassette were able to grow on fucose (Fig 2), while some strains lacking this cassette did grow on fucose, indicating that additional genes may be involved in fucose utilization.

Four *B*. *cereus* strains were found to grow on mannose, i.e strains B4077, B4081, B4087, and B4120 (Fig 2). A mannose utilization cassette *manRPA* is present in genomes of three of those tested strains (B4077, B4081 and B4087) and in genomes of *B*. *cereus* strains 03BB102, F837_76, Q1 and in *B*. *subtilis* 168. The cassette encodes a mannose-specific PTS transporter ManP, a mannose 6P isomerase ManA and a BglB family transcriptional antiterminator ManR (S2 Table). The *manRPA* cassette, as well as any other gene cassette annotated as “mannose” utilization, was not found in strain B4120.

A 9-gene cassette *iolRTGCADEJB* for myo-inositol utilization, orthologous to that of *B*. *subtilis* 168, is present in three of the 22 experimentally verified strains B4077, B4082, B4083 and in genomes of *B*. *cereus* reference strains 03BB102, AH820, BCM2_134A, CI, E33L (with an additional plasmid-located cassette), F837_76 and FRI_35. None of the strains grew on inositol in the API test, but 11 strains were found to grow in the aerobic growth experiment in defined medium (Fig 2) including two strains harbouring the *iol* cassette (i.e. B4077, B4082). Therefore there must be an additional cassette for inositol utilization.

Additionally, 8 strains were observed to grow on N-acetyl-galactosamine in aerated medium, while 3 strains showed possible growth (Fig 2). The annotation of most of the genes in a 10-gene cassette (BCE1899-BCE1908 of strain ATCC 10987), previously suggested to be related to growth on tagatose [31], suggests that the actual substrate could be N-acetyl-galactosamine. The cassette encodes a 3-component ABC transporter (putatively for N-acetylhexosamines; OG_9457, OG_6509, OG_6510), and all enzymes required for degradation of N-acetyl-galactosamine via the tagatose pathway to glyceraldehyde-3P (**Table B in** S2 Table) [63]. However, we find this complete gene cassette only in *B*. *cereus* strains B4079, ATCC 10987, E33L, and FRI_35 which does not correlate well with observed growth on N-acetyl-galactosamine. Nevertheless none of the strains grow on tagatose in static conditions (Fig 2), therefore it seems unlikely that this gene cluster is related to growth on tagatose. We found another 14-gene cassette presumably for utilization of the disaccharide Gal-(1->3)-beta-GalNAc (galactose linked to N-acetyl-galactosamine) (**Table B in** S2 Table). Gal-GalNAc is bound to proteins in mucins (O-linked to Ser/Thr), and can be released by extracellular endo-alpha-N-acetylgalactosaminidases. This cassette is very similar to the cassette described above for utilization of N-acetyl-galactosamine, but it has 2 additional genes encoding extracellular endo-alpha-N-acetyl-galactosaminidases, and a gene encoding an intracellular 1,3-beta-galactosyl-N-acetylhexosamine phosphorylase which cleaves Gal-(1->3)-beta-GalNAc into alpha-D-galactose-1P and N-acetyl-D-galactosamine. This cassette is found in 8 of 22 experimentally verified *B*. *cereus* strains and two reference genomes (Q1 and NC7401) (S3 Table), but it could not explain utilisation of Gal-GalNAc nor N-acetyl-galactosamine by strains B4083, B4087, B4118, and B4147.

Recently, a new sugar phosphate uptake system specific for glucose-6-phosphate and fructose-6-phosphate has been described in *B*. *cereus* [64]. This 5-gene cassette includes a transporter SpsABC and a two-component sensor system SpsKR which responds to extracellular sugar phosphate levels. The *spsA* gene was specifically expressed during oral infection of *Galleria mellonella* [65]. The cassette was found in all the *B*. *cereus* genomes, and all but three (B4083, B4117, ATCC 14579) experimentally validated strains were observed to grow on glucose-6-phosphate (Fig 2).

#### Other carbohydrates

In *B*. *cereus* genomes there are several putative gluconate uptake and utilization systems, based on the gluconate permease GntP. The annotation of these systems suggests that the substrate is gluconate, but it could also be glucuronate or galacturonate, or derivatives of these acids. Orthologs of the gluconate utilization gene cassette *gntRKPZ* of *B*. *subtilis* 168 [66] were found in 8 of 22 experimentally verified strains and in additional 8 genomes (S3 Table). All but one (ATCC 10987) experimentally validated strains belonging to phylogenetic group III carried this cassette encoding a gluconate permease GntP, a gluconate kinase GntK, a 6-phosphogluconate dehydrogenase GntZ, and a gluconate operon repressor GntR. A second cassette (system II), not present in *B*. *subtilis* or *B*. *anthracis*, is the same as system I but lacks the regulator, and is present in 16 out of 22 experimentally validated strains, mainly not belonging to phylogenetic group III, and five additional genomes (S3 Table). A third cassette (system III) with a gluconate permease GntP is present in all considered *B*. *cereus sensu lato* group genomes, but not in *B*. *subtilis*. This cassette also encodes a GntR family transcription regulator, a 2-keto-3-deoxygluconate kinase, an amidohydrolase, an aldolase and a pyridoxal phosphate-dependent enzyme, suggesting that this cassette may encode utilization of a derivative of gluconate such as D-glucosaminate. Finally, a fourth 7-gene cassette (system IV) is present in all *B*.*cereus* genomes, and next to the *gntP*, *gntK* and *gntZ* genes it contains genes encoding 6-phosphogluconolactonase Pgl, glucose-6-phosphate 1-dehydrogenase, transketolase, and transaldolase (**Table C in** S2 Table). However, in a few strains, i.e. *B*. *cereus* strains B4083, B4088, B4117, and G9842, this cassette lacks both the *gntP* and transketolase genes, suggesting that this cassette represents a breakdown pathway of glucose-6P via gluconate-6P, and not an uptake system for gluconate.

None of the strains grew on potassium gluconate in the API test, but in the growth experiments with aeration most strains showed growth (Fig 2) suggesting that metabolism of this carbohydrate requires oxygen. Only strains B4083, B4088, and B4155 showed poor or no growth on gluconate. This phenotype correlates best with the gluconate cassette system IV, and therefore this cassette may represent the main gluconate utilization system under these growth conditions.

Arbutin, esculin and salicin are aryl beta-glucosides (or aromatic beta-glucosides), consisting of a glucose moiety linked to an aromatic ring. The *B*. *subtilis* 168 genome encompasses 3 genes encoding intracellular (aryl-) phospho-beta-D-glucosidases, i.e. *bglA* (BSU40110), *bglC/yckE* (BSU03410), and *bglH* (BSU39260) [67]. In the *B*. *cereus* genomes there is no equivalent of *bglA* of *B*. *subtilis*. The *bglC* gene (OG_4907) of *B*. *subtilis* 168 has one ortholog in 11 *B*. *cereus* strains, but it is not part of a sugar utilisation cassette, while a second ortholog of *bglC* is found only in strains B4082 and AH820 as part of a 7-gene cassette for cellobiose utilization. Finally, *B*. *cereus* strains B4077, B4083, B4088, B4117, E33L and FRI_35 carried orthologues of *B*. *subtilis* 168 *bglPH* operon encoding a beta-glucoside-specific PTS system BglP, and an aryl-phospho-beta-D-glucosidase or aryl-6-phospho-beta-glucosidase BglH [68] additionally encoding also a beta-glucoside *bgl* operon antiterminator BglG. Taken together, there are 14 *B*. *cereus* strains that have 1 or 2 genes encoding an aryl-6-phospho-beta-glucosidase. It is not clear what the transporter is for aryl beta-glucosides in these strains, but it is possibly a more general PTS for beta-glucosides, such as for cellobiose (see above for 3 putative PTS cellobiose utilization cassettes). This occurrence of one or more aryl-6-phospho-beta-glucosidases in 12 *B*. *cereus* strains correlates almost perfectly with the growth observed on salicin (Fig 2). The only discrepancies correspond to uncertainties in the API test. On the other hand, there are 20 strains that grow on arbutin and esculin, suggesting that there may be an additional system in the other strains which is more specific for arbutin and esculin, but not for salicin.

None of the strains grew on glycerol in the API test, but in the growth experiments with aeration all but three (B4077, B4085, and ATCC 10987) strains showed growth (Fig 2). A glycerol utilization cassette, equivalent to the *glpPFKD* genes (BSU09270-BSU09300) of *B*. *subtilis* 168, was found to be present in all *B*. *cereus* genomes. The cassette encodes a glycerol uptake facilitator protein GlpF, a glycerol kinase GlpK, an aerobic glycerol-3-phosphate dehydrogenase GlpD, and a glycerol uptake operon regulator Glp (**Table C in** S2 Table). Therefore all strains have the potential to grow on glycerol in aerobic conditions.

A glycerol-phosphate uptake system, based on an ABC transporter UgpBEAC, was found in most of *B*. *cereus* genomes, but not in strains B4082, B4083, B4088, B4147, 03BB102, and F837_76. Moreover, all *B*. *cereus* genomes encode a glycerol-3-phosphate permease (OG_511). However this substrate was not experimentally tested.

#### Absent sugar utilization systems

There are a few sugar utilization cassettes of *B*. *subtilis* 168 which did not have ortholog cassettes in any of the *B*. *cereus* strains. These include the mannitol cassette *mtlAFD* (BSU03981, BSU03982, BSU03900), the sorbitol/glucitol cassette *gutRBP-fruC* (BSU06140-BSU06170), the arabinan/arabinose cassette *abnA-araABDLMNPQ-apfA* (BSU28720-BSU28810), the L-rhamnose cassette *rhaAMBR(EW)* (BSU31180-BSU31220), and the oligomannoside cassette *gmuBACDREFG* (BSU05810-BSU05880). In agreement with this lack of genes, the selected *B*. *cereus* strains in our study did not grow on mannitol, sorbitol, arabinose or rhamnose (Fig 2).

Orthologs of the levan/fructose-specific PTS system *levDEFG* of *B*. *subtilis* 168 are only found in *B*. *cereus* strain E33L on a plasmid. However, the cassette in E33L is much larger (12 genes) and may be specific for breakdown of more complex polysaccharides as levan is a fructose polymer (fructan).

## Discussion

Equipment of ubiquitous food-borne human pathogens such as *B*. *cereus* with a broad range of carbohydrate transporter and utilisation systems supports their transmission from soil to host. Comparative analysis of genotypic and phenotypic diversity of carbohydrate utilisation among 20 newly sequenced *B*. *cereus* food isolates and *B*. *cereus* ATCC 14579 and ATCC 10987 suggests an adaptative metabolic capacity to different environmental niches represented by carbohydrates such as cellobiose or xylose found in soil, starch or lactose in food and dairy products, and fucose and glucose-6-phosphate found in intestinal tracts of animal and human hosts. In addition to such strain-specific features, all the strains could utilise compounds typically reported to support growth of *B*. *cereus* such as glucose, fructose, maltose, trehalose, N-acetyl-glucosamine, and ribose [23,69,70].

The diversity in sugars utilised by *B*. *cereus* isolates may correspond to the classification of the isolates to four of the phylogenetic groups identified previously by Guinebretière [22] within the *B*. *cereus* sensu lato group. For example strains in groups VI and II show a broader substrate utilisation capacity compared to strains belonging to group III. Members of the latter group, with exception of strain B4087, did not grow on glycogen, starch or salicin. In addition, the genomes of these strains lack aryl-6-phospho-beta-glucosidases involved in the utilisation of two other aromatic glucosidases, esculin and arbutin. Notably, strain B4087 displayed a distinctive growth pattern and genome potential compared to the other members of group III. Within group IV, a sub-branch encompassing strains isolated from vegetables (B4080, B4084, B4158), showed limited substrate utilisation. Interestingly, two strains (B4079 and ATCC 10987) representing a sub- branch within group III showed the least carbohydrate utilisation capacity, and both strains originate from spoilage incidents, namely from a retorted can with chocolate beverage and spoiled cheese, respectively.

**Fig 4:**
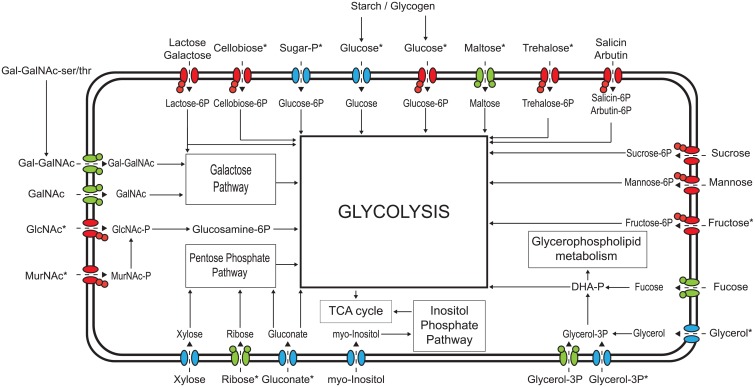
Schematic overview of predicted carbohydrate utilization systems in *B*. *cereus*. For transporters, green indicates a putative ABC transporter; red, a putative PTS transporter; and blue, a putative permease. Star indicates systems present in all 22 strains. Abbreviations used are N-Acetyl-glucosamine (GlcNAc), N-Acetyl-galactosamine (GalNAc), galactose-N-Acetyl-galactosamine (Gal-GalNAc), N-Acetyl-muramic acid (MurNAc), Dihydroxyacetone phosphate (DHA-P).

Carbohydrate uptake in bacteria is mediated by different transport mechanisms including phosphoenol-pyruvate-dependent phosphotransferase systems (PTS systems), ATP-binding transporters (ABC transporters) and secondary transporters (permeases). We searched for transporters and associated metabolic enzymes in the genomes of newly sequenced *B*. *cereus* isolates and identified a significant number of new carbohydrate utilisation cassettes. The collective capacity of *B*. *cereus* strains to utilise carbohydrates is visualised in Fig 4. Carbohydrate uptake in bacilli occurs mainly by PTS systems as indicated by identification of 15 PTS cassettes in *B*. *cereus* genomes (see worksheet PTS systems in S3 Table), while ABC transporters and permeases for carbohydrate uptake contribute with 6 and 11 identified systems, respectively (see worksheet ABC-permeases in S3 Table).

The possible roles of specific carbohydrate transporter and utilisation systems in transmission of *B*. *cereus* from soil, via food processing environments and food, to the host, is discussed below.

The soil environment is rich in chitin, a constituent of insects exoskeletons and cuticles but also fungal cell walls. Chitin is a 1–4 polymer of N-acetyl-glucosamine and by activity of commonly found chitinases, simple amino sugars can be released and become available for soil bacteria [3]. Amino sugars such as N-acetyl-glucosamine and N-acetyl-muramic acid are also the major components of bacterial peptido-glycan. The presence of *nagEABR* and *murRQP* cassettes in all tested isolates perfectly correlates with the observed growth on these substrates.

Cellobiose is a catabolite of cellulose degradation, and is therefore also present in soil and decaying vegetation. While all the *B*. *cereus* isolates possess three or four cassettes for cellobiose utilisation, only five *B*. *cereus* isolates were found to utilise cellobiose in static conditions, which suggests that activation of cellobiose systems is not achieved in most cases. Conceivably, activation of cellobiose uptake and utilisation systems requires specific conditions that remain to be elucidated. In *Listeria monocytogenes* presence of β-glucosides (cellobiose, salicin but not arbutine) in the environment causes repression of virulence genes via a specific regulatory pathway (involving *bvr* locus) independent from carbon catabolite repression (CCR) mechanism [71,72] as well as via a CCR mechanism. A specific role for cellobiose in repression of *B*. *cereus* enterotoxin production has not been reported up to now.

Notably, growth on xylose, another common plant-derived carbohydrate was only observed for *B*. *cereus* ATCC 10987, and this perfectly matched the unique presence of the previously described cassette for xylose utilisation in this strain [28].

Concerning transport and utilisation systems related to food, the capacity to grow on lactose, a common carbohydrate in milk and dairy environment, is restricted to only two of 22 tested *B*. *cereus* strains. This is in line with previous observations, that showed limited ability to utilise lactose among *B*. *cereus* dairy farm isolates, although prevalence increased along the production chain, reaching 20% lactose-positive strains among *B*. *cereus* household milk isolates [47], pointing to the selection and adaptation of the strains in the dairy chain. The two lactose-positive strains in our study contain a lactose utilisation cassette predicted to be on a plasmid. This lactose cassette is also present in genomes of a few other *B*. *cereus* strains not included in this study (S4 Table). Similarity with cassettes present in other bacteria is limited, suggesting that mobility of this *B*. *cereus* specific lactose cassette may be responsible for the increasing prevalence of lactose-positive strains.

Notably, *B*. *cereus* potential to growth on starch was found to depend on the presence of a single gene encoding alpha-amylase, AmyS. This extracellular enzyme degrades starch releasing glucose that can be transported and utilised by one of the glucose uptake systems commonly found in *B*. *cereus* strains.

Concomitant with milk or other food products *B*. *cereus* can enter the host gastro-intestinal tract and encounter a nutrient-limited and competitive environment. One of the available substrates is fucose, a major component of mucin glycoproteins. Fucose is abundant in the human intestine [73] and is often associated with biologically active molecules such as serum glycoproteins, immunoglobulins, blood group substances, or gastric and submaxillary mucins [61]. Of the 22 tested *B*. *cereus* strains, seven contained a *fcs* fucose utilisation cassette in their genomes, whereas 12 strains showed growth or poor growth on fucose, suggesting the presence of an additional or alternative system. The Fcs system includes extracellular α-L-fucosidase that may liberate fucose from natural substrates e.g. host mucin, but not always from artificial substrates such as p-nitrophenyl or α-L-fucoside as used in our assay. However, several soil isolated Bacilli are able to utilise both categories of substrates by producing more types of α-L-fucosidases [60–62]. Although fucose and the *fcs* system influence *Streptococcus pneumoniae* virulence [74] and the human gut microbe *Bacteroides thetaiotaomicron* can harvest monomeric fucose from host Fucα1,2-Galβ-containing structures [73], the role of the Fcs system in *B*. *cereus* performance in the intestine and/or pathogenicity remains to be elucidated.

A disaccharide Gal-GalNAc (galactose linked to N-acetyl-galactosamine) is also bound to proteins in mucins. Prior to uptake and utilisation these mucin-bound sugars can be released by endo-alpha-N-acetylgalactosaminidases which are generally found in the human gut. Notably, 8 of the tested *B*. *cereus* strains encode this extracellular enzyme. The cassette for Gal-GalNAc utilisation possess also the genes required for utilisation of N-acetyl-galactosamine. Theoretically if GalNAc would be transported into the cell, it could be utilised by those 8 strains. However, that still does not explain growth of 11 of the tested *B*. *cereus* strains on GalNAc. Interestingly all but one of the strains utilising GalNAc could also use inositol, and the majority of them could use fucose as well suggesting their adaptation to the gut environment.

Finally, 19 of tested *B*. *cereus* strains were shown to utilise glucose-6-phosphate, while genomes of all the strains carry the conserved sugar-phosphate specific *spsRKABC* cassette [64]. Sugar phosphates can be found in a range of hosts including the perithropic matrix separating food bolus from the mid-gut epithelium cells of insects, possibly facilitating initial multiplication of bacteria in the gut and their persistence, an initial step for host infection [75]. In particular the Sps system was shown to play a role in *B*. *cereus* sugar phosphate sensing and uptake during growth in the intestinal environment of *Galleria mellonella* larvae [64]. Moreover, glucose-6-phosphate was shown to inhibit expression of *hlyII*, encoding the pore-forming toxin haemolysin II, by activation of HlyIIR in plasmid-curated acrystalliferous *B*. *thuringiensis* [32].

Besides the effect of available carbohydrates on the expression of virulence factors, that may facilitate the host infection as well as outcompeting competitive flora, carbohydrates were shown to affect biofilm formation both in gram- positive and gram-negative bacteria [76–78]. Formation of biofilms can facilitate colonisation of environmental niches including soil, food-processing environments and host intestine [79]. Roles of the different carbohydrates in *B*. *cereus* biofilm formation and virulence remain to be established.

The analysis of complete carbohydrate transporter and utilization cassettes allowed for the prediction of substrate utilization. Particularly for carbohydrate substrates used by only small numbers of strains such as xylose, lactose, mannose, sucrose, starch/glycogen, and salicin, there is an excellent correlation with the presence of the corresponding gene cassettes. Discrepancies between observations and predictions are conceivably due to: i. lack of expression of cassettes in tested conditions, e. g. availability of oxygen, as the number of strains utilising gluconate, L-fucose and inositol was higher in defined, aerated media compared to static API tests; ii. miss-identification of cassettes based on incorrect or incomplete prediction of carbohydrate specificity of transporters; and iii. lack of identification of cassettes e.g. presence of several ABC transporters with unknown specificity and proteins annotated only as “membrane proteins” may function as transporters (e.g. permeases). The latter two points of the genome-mining approach are clearly limitations for the identification of novel sugar utilization pathways which have not been described in literature earlier.

In conclusion, this study provides extensive information on the genetic potential of *B*. *cereus* strains as well as the corresponding potential to utilise carbohydrates for growth, reflecting strain diversity and the capacity to occupy different niches including soil, food products as well as intestinal tract of insects and mammals.

## Supporting Information

S1 Table Metadata on *Bacillus* strains and genomes. (PDF)

S2 Table Summary of carbohydrate utilization cassettes and encoded functions. (A) PTS system cassettes, (B) ABC transporter cassettes, (C) permease cassettes. * genes with newly predicted function.
(PDF)

S3 Table Annotation of carbohydrate utilization cassettes and their genes in *B*. *cereus* genomes. The information in each cell represents: NCBI accession code or contig number (NODE/scaffold) | locus tag | nt coordinates | number of encoded amino acids. Blue shading indicates putative pseudogenes. * genes with newly predicted function.
(XLSX)

S4 Table *B*. *cereus* lactose cassette homology to other species and strains. (PDF)
