# Supplementary material for: Linking Bacillus cereus Genotypes and Carbohydrate Utilization Capacity
Source: PLoS One. 2016 Jun 7;11(6):e0156796. doi: 10.1371/journal.pone.0156796 (PMC4896439; doi:10.1371/journal.pone.0156796)
Supplement: S1 Table — (PDF) [file pone.0156796.s001.pdf]

S1 Table. Metadata on *Bacillus* strains and genomes

Category	Strain	Isolation source /origin	Used for*	Assembly / WGS code or UID
**New *Bacillus cereus* genomes**	Bacillus_cereus_B4077	Chilled dessert	P, E, G	LCYI00000000.1
	Bacillus_cereus_B4078	Food, unknown	P, E, G	LCYJ00000000.1
	Bacillus_cereus_B4079	Canned chocolate beverage	P, E, G	LJIT00000000
	Bacillus_cereus_B4080	Dried onion	P, E, G	LCYK00000000.1
	Bacillus_cereus_B4081	Provolone sauce	P, E, G	LJJZ00000000
	Bacillus_cereus_B4082	Asparagus ham sauce	P, E, G	LJKA00000000
	Bacillus_cereus_B4083	Torteloni con fughi	P, E, G	LJKB00000000
	Bacillus_cereus_B4084	Indian rice dish	P, E, G	LJKC00000000
	Bacillus_cereus_B4085	Asparagus soup	P, E, G	LJKD00000000
	Bacillus_cereus_B4086	Boiled rice	P, E, G	LCYL00000000.1
	Bacillus_cereus_B4087	Pea soup	P, E, G	LCYM00000000.1
	Bacillus_cereus_B4088	Dressing	P, E, G	LJKE00000000
	Bacillus_cereus_B4116	White sauce	P, E, G	LJKF00000000
	Bacillus_cereus_B4117	Commercial pasteurised milk	P, E, G	LJKG00000000
	Bacillus_cereus_B4118	Ice cream	P, E, G	LJKH00000000
	Bacillus_cereus_B4120	Water	P, E, G	LJKI00000000
	Bacillus_cereus_B4147	Cereals, pasta and pastries	P, E, G	LCYN00000000.1
	Bacillus_cereus_B4153	Dairy products	P, E, G	LCYO00000000.1
	Bacillus_cereus_B4155	Beef salad	P, E, G	LJKJ00000000
	Bacillus_cereus_B4158	Vegetables	P, E, G	LCYP01000000
***Bacillus cereus* reference genomes**	Bacillus_cereus_ATCC14579	Unknown; Type Strain	P, E, G	uid57975
	Bacillus_cereus_ATCC10987	Cheese spoilage	P, E, G	uid57673
	Bacillus_cereus_03BB102	Blood of human fatal pneumonia	P, G	uid59299
	Bacillus_cereus_AH187	Vomit of a person; emetic outbreak	P, G	uid58753
	Bacillus_cereus_AH820	Human periodontal pocket	P, G	uid58751
	Bacillus_cereus_B4264	Blood of human; fatal pneumonia	P, G	uid58757
	Bacillus_cereus_biovar_anthracis_CI	Lethal anthrax in chimpanzee	P, G	uid50615
	Bacillus_cereus_E33L	Isolated from a dead zebra carcass	P, G	uid58103
	Bacillus_cereus_F837/76	Prostate wound	P, G	uid83611
	Bacillus_cereus_FRI-35	Unknown	P, G	uid173403
	Bacillus_cereus_G9842	Human stool	P, G	uid58759
	Bacillus_cereus_NC7401	Emetic-type food poisoning	P, G	uid82815
	Bacillus_cereus_Q1	Deep-subsurface oil reservoir	P, G	uid58529
**Other reference genomes**	Bacillus_subtilis_168	Unknown; Type Strain	P, G	uid57675
	Bacillus_weihenstephanensis_KBAB4	Soil isolate	P	uid58315
	Bacillus_thuringiensis_Bt407	Unknown	P	uid177931
	Bacillus_thuringiensis_MC28	Soil isolate	P	uid176369
	Bacillus_thuringiensis_HD771	Unknown	P	uid173374
	Bacillus_thuringiensis_HD789	Unknown	P	uid173860
	Bacillus_thuringiensis_BMB171	Acrystalliferous mutant of biopesticide strain	P	uid49135
	Bacillus_thuringiensis_Al_Hakam	Suspected bioweapons facility	P	uid58795
	Bacillus_thuringiensis_serovar_IS5056	Soil isolate	P	uid190186
	Bacillus_thuringiensis_serovar_finitimus_YBT020	Unknown	P	uid158875
	Bacillus_thuringiensis_serovar_kurstaki_HD73	Unknown	P	uid189188
	Bacillus_thuringiensis_serovar_chinensis_CT43	agricultural biopesticide	P	uid158151
	Bacillus_thuringiensis_serovar_konkukian_97_27	Severe human tissue necrosis	P	uid58089
	Bacillus_anthracis_Ames	Texas, Cow; plasmids cured	P	uid57909
	Bacillus_anthracis_Ames_Ancestor	Texas, Cow; plasmids included	P	uid58083
	Bacillus_anthracis_Sterne	Unknown	P	uid58091
	Bacillus_anthracis_A0248	Human isolate	P	uid59385
	Bacillus_anthracis_H9401	Human isolate	P	uid162021
	Bacillus_anthracis_CDC684	Human isolate	P	uid59303

***Strain used for (P)hylogeny, (E)xperiments, and (G)enom comparison**
