# Supplementary material for: Linking Bacillus cereus Genotypes and Carbohydrate Utilization Capacity
Source: PLoS One. 2016 Jun 7;11(6):e0156796. doi: 10.1371/journal.pone.0156796 (PMC4896439; doi:10.1371/journal.pone.0156796)
Supplement: S2 Table — (A) PTS system cassettes, (B) ABC transporter cassettes, (C) permease cassettes. * genes with newly predicted function. (PDF) [file pone.0156796.s002.pdf]

S2 Table. Summary of carbohydrate utilization cassettes and encoded functions. (A) PTS system cassettes, (B) ABC transporter cassettes, (C) permease cassettes. * genes with newly predicted function

A. PTS system cassettes

Carbohydrate	OG	Gene	Predicted function
**Fructose**	OG_788	fruA	PTS system, fructose-specific IIABC component
	OG_787	fruK	1-phosphofructokinase (EC 2.7.1.56)
	OG_786	fruR	transcriptional regulator, DeoR family
**Glucose**	OG_767	ptsI	phosphotransferase system (PTS) enzyme I
	OG_1852	hpr	phosphotransferase system, phosphocarrier protein HPr
	OG_167	ptsG	PTS system, glucose-specific subunit IICBA
	OG_766	glcT	transcriptional antiterminator, BglG family
**Trehalose**	OG_568	treR	transcriptional regulator, GntR family
	OG_567	treB	PTS system, trehalose-specific IIBC component
	OG_10	treC*	trehalose-6-phosphate hydrolase (EC 3.2.1.93)
**N-acetylglucosamine**	OG_565	nagE	PTS system, N-acetylglucosamine-specific IICB component
	OG_1594	nagR	transcriptional regulator, GntR family
	OG_1595	nagB	glucosamine-6-phosphate deaminase (EC 3.5.99.6)
	OG_1596	nagA	N-acetylglucosamine-6-phosphate deacetylase (EC 3.5.1.25)
**N-acetyl muramic acid**	OG_2360	murR	transcriptional regulator, RpiR family
	OG_267	murQ	N-acetylmuramic acid 6-phosphate etherase (EC 4.2.-.-)
	OG_451	murP	PTS system, N-acetylmuramic acid-specific IIBC component
**mannose**	OG_222	manB*	phosphomannomutase (EC 5.4.2.8)
	OG_5605	manR	activator of the mannose operon, BglG family
	OG_5606	manP	PTS system, mannose-specific IIBCA component
	OG_5607	manA	mannose-6-phosphate isomerase (EC 5.3.1.8)
**sucrose**	OG_4398	scrK	fructokinase (EC 2.7.1.4)
	OG_4407	scrB	sucrose-6-phosphate hydrolase (EC 3.2.1.26)
	OG_4406	scrA	PTS system, sucrose-specific enzyme IIBC component
	OG_4581	scrR	sucrose operon repressor
**(aryl)beta-glucosides**	OG_5776	bglG	beta-glucoside bgl operon antiterminator, BglG family
	OG_5777	bglP	PTS system, beta-glucoside-specific IIBCA component
	OG_5282	bglH	aryl-phospho-beta-d-glucosidase/ 6-phospho-beta-glucosidase
**alpha-glucosides**	OG_10	agl	alpha-glucosidase (EC 3.2.1.20)/oligo-1,6-glucosidase (EC 3.2.1.10)
	OG_2534	aglP	PTS system, maltose and glucose-specific IIBC component
	OG_1043	aglA	PTS system, glucose-specific IIA component
**cellobiose I**	OG_1960	bglG	transcription antiterminator, BglG family
	OG_2618	celA	PTS system, cellobiose-specific IIA component
	OG_2362	celB	PTS system, cellobiose-specific IIB component
	OG_2619	celC	PTS system, cellobiose-specific IIC component
**cellobiose II**	OG_3271	ydjC	cellobiose PTS system YdjC-like protein
	OG_1728	bglA	6-phospho-beta-glucosidase
	OG_2482	celA	PTS system, cellobiose-specific IIA component
	OG_3272	celC	PTS system, cellobiose-specific IIC component
	OG_2361	celB	PTS system, cellobiose-specific IIB component
**cellobiose III**	OG_1728	licH	6-phospho-beta-glucosidase
	OG_1729	licA	lichenan-specific PTS system, IIA component
	OG_171	licC	PTS system, cellobiose-specific IIC component
	OG_1730	licB	lichenan-specific PTS system, IIB component
	OG_1960	licR	transcriptional antiterminator of lichenan operon, BglG family
**cellobiose IV**	OG_4907	bglC2	6-phospho-beta-glucosidase (EC 3.2.1.86)
	OG_12395	celA	PTS system, cellobiose-specifict subunit IIA
	OG_12396	celC	PTS system, cellobiose-specifict subunit IIC
	OG_12397	pgm	phosphoglycerate mutase
	OG_12398	-	hydrolase
	OG_7868	celB	PTS system, cellobiose-specific IIB component
	OG_12399	merR	transcriptional regulator, MerR family
**lactose**	OG_5826	lacD	tagatose 1,6-bisphosphate aldolase (EC 4.1.2.40)
	OG_5477	lacC	tagatose-6-phosphate kinase (EC 2.7.1.144)
	OG_12576	lacB	galactose-6-phosphate isomerase, LacB subunit (EC 5.3.1.26)
	OG_12575	lacA	galactose-6-phosphate isomerase, LacA subunit (EC 5.3.1.26)
	OG_9651	lacR	lactose PTS system repressor, DeoR family
	OG_9650	lacF	PTS system, lactose-specific IIA component
	OG_9649	lacE	PTS system, lactose-specific IIBC component
	OG_4907	lacG	6-phospho-beta-galactosidase (EC 3.2.1.85)
	OG_12574	glk	glucokinase (EC 2.7.1.2)/transcriptional repressor, ROK family

B. ABC transporter cassettes

Carbohydrate	OG	Gene	Predicted function
**maltose/maltodextrin**	OG_2407	malR	maltose operon transcriptional repressor, LacI family
	OG_2085	malG	maltose/maltodextrin ABC transporter, permease protein
	OG_2084	malF	maltose/maltodextrin ABC transporter, permease protein
	OG_2212	malE	maltose/maltodextrin ABC transporter, substrate-binding protein
	OG_2083	amyA	neopullulanase (EC 3.2.1.135)/alpha-amylase
	OG_10	malL	alpha-glucosidase (EC 3.2.1.20)/oligo-1,6-glucosidase (EC 3.2.1.10)
	OG_91	malK	maltose/maltodextrin ABC transporter, ATPase (EC 3.6.3.19)
**Ribose**	OG_1681	rbsR	ribose operon transcriptional regulator, LacI family
	OG_1680	rbsK	ribokinase (EC 2.7.1.15)
	OG_1971	rbsD	ribose ABC transport system, permease protein RbsD
	OG_1679	rbsA	ribose ABC transport system, ATP-binding protein RbsA
	OG_1678	rbsC	ribose ABC transport system, permease protein RbsC
	OG_1957	rbsB	ribose ABC transport system, ribose-binding protein RbsB
	OG_1632	tal	transaldolase (EC 2.2.1.2)
**Fucose**	OG_6050	alfA*	alpha-L-fucosidase, glycosyl hydrolase family 98 (EC 3.2.1.51)
	OG_5773	aldA*	lactaldehyde dehydrogenase (EC 1.2.1.22)
	OG_6212	fcsI	L-fucose isomerase (EC 5.3.1.25)
	OG_5772	fcsK*	L-fuculokinase (EC 2.7.1.51)
	OG_6211	fcsA	L-fuculose phosphate aldolase (EC 4.1.2.17)
	OG_6210	fcsB*	fucose ABC transporter,substrate-binding component
	OG_6209	fcsC*	fucose ABC transporter, permease component
	OG_6208	fcsD*	fucose ABC transporter, ATP-binding protein
	OG_6207	fcsU*	L-fucose mutarotase/D-ribose pyranase
	OG_6206	fcsR*	transcriptional regulator, LacI family
**glycerol-3-phosphate**	OG_4036	ugpR	glycerol uptake operon antiterminator regulatory protein
	OG_4037	ugpC	glycerol-3-phosphate ABC transporter, ATP-binding protein
	OG_3605	ugpA	glycerol-3-phosphate ABC transporter, permease
	OG_2005	ugpE	glycerol-3-phosphate ABC transporter, permease
	OG_3358	ugpB	glycerol-3-phosphate ABC transporter, substrate-binding protein
**N-acetyl-galactosamine**	OG_9457	agaB*	N-Acetyl-hexosamine ABC transporter, sugar-binding protein
	OG_6509	agaC*	N-Acetyl-hexosamine ABC transporter, permease protein
	OG_6510	agaD*	N-Acetyl-hexosamine ABC transporter, permease protein
	OG_7068	mviM	oxidoreductase family protein
	OG_7035	agaR	transcriptional regulator, GntR family
	OG_5649	agaS	galactosamine-6-phosphate isomerase (EC5.3.1.-)
	OG_5650	agaA	N-acetylglucosamine-6-phosphate deacetylase (EC3.5.1.25)
	OG_9456	agaY	tagatose 1,6-bisphosphate aldolase (EC 4.1.2.40)
	OG_5477	agaZ	tagatose-6-phosphate kinase (EC 2.7.1.144)
	OG_9455	agaK*	sugar kinase, ROK family
**Gal-GalNAc**	OG_6064	araC	transcriptional regulator, AraC family
	OG_6065	agaB*	N-Acetyl-D-hexosamine ABC transporter, sugar-binding protein
	OG_5526	agaC*	N-Acetyl-D-hexosamine ABC transport system,permease
	OG_6000	agaD*	N-Acetyl-D-hexosamine ABC transport system,permease
	OG_6066	lnbP	1,3-beta-galactosyl-N-acetylhexosamine phosphorylase (EC 2.4.1.211)
	OG_6226	-	hypothetical protein
	OG_6067	agaK*	N-acetylhexosamine-6P-responsive transcriptional repressor
	OG_5649	agaS*	galactosamine-6-phosphate isomerase (EC5.3.1.-)
	OG_5650	agaA	N-acetylgalactosamine-6-phosphate deacetylase (EC3.5.1.25)
	OG_5477	agaY	tagatose-6-phosphate kinase (EC 2.7.1.144)
	OG_5826	agaZ	tagatose 1,6-bisphosphate aldolase (EC 4.1.2.40)
	OG_5120	agaM1*	endo-alpha-N-acetylgalactosaminidase (3.2.1.97), extracellular
	OG_5120	agaM2*	endo-alpha-N-acetylgalactosaminidase (3.2.1.97), extracellular
	OG_5439	galT	galactose-1-phosphate uridylyltransferase (EC 2.7.7.10)

C. permease cassettes

Carbohydrate	OG	Gene	Predicted function
**myo-inositol**	OG_4967	iolB	5-deoxy-glucuronate isomerase (EC 5.3.1.30)
	OG_5360	iolJ*	6-phospho-5-dehydro-2-deoxy-D-gluconate aldolase (EC 4.1.2.29)
	OG_5286	iolE	inosose dehydratase (EC 4.2.1.44)
	OG_5017	iolD*	3D-(3,5/4)-trihydroxycyclohexane-1,2-dionehydrolase (EC 3.7.1.22)
	OG_85	iolA	methylmalonate-semialdehyde dehydrogenase [inositol] (EC 1.2.1.27)
	OG_5016	iolC	5-keto-2-deoxygluconokinase (EC 2.7.1.92)
	OG_4618	iolG	myo-inositol 2-dehydrogenase (EC 1.1.1.18)
	OG_4851	iolT*	polyol transporter, MFS superfamily
	OG_4904	iolR	transcriptional regulator, LacI family
**xylose**	OG_8153	xylR	xylose-responsive transcription regulator, ROK family
	OG_8154	xylA	xylose isomerase (EC 5.3.1.5)
	OG_7412	xylB	xylulose kinase (EC 2.7.1.17)
	OG_12863	xylE*	xylose 1-epimerase
	OG_2079	xylP	xylose permease
**sugar phosphate**	OG_3282	spsC*	sugar phosphate permease, MFS superfamily
	OG_3283	spsB	transporter, sustrate-binding protein
	OG_3284	spsA*	membrane-anchored protein
	OG_3285	spsK	two-component system, sensor histidine kinase
	OG_3286	spsR	two-component system, response regulator
**glycerol**	OG_619	glpP	glycerol uptake operon antiterminator regulatory protein
	OG_620	glpF	glycerol uptake facilitator protein
	OG_621	glpK	glycerol kinase (EC 2.7.1.30)
	OG_622	glpD	aerobic glycerol-3-phosphate dehydrogenase (EC 1.1.5.3)
**glucose**	OG_273	Gdh	glucose 1-dehydrogenase (EC 1.1.1.47)
	OG_274	glcU	glucose uptake protein
**gluconate-I**	OG_4734	gntR	gluconate operon transcriptional repressor
	OG_45	gntK	gluconokinase (EC 2.7.1.12)
	OG_247	gntP	gluconate permease
	OG_4493	Gnd	6-phosphogluconate dehydrogenase, decarboxylating (EC 1.1.1.44)
**gluconate-II**	OG_45	gntK	gluconokinase (EC 2.7.1.12)
	OG_247	gntP	gluconate permease
	OG_4868	Gnd	6-phosphogluconate dehydrogenase,decarboxylating (EC 1.1.1.44)
**gluconate-III**	OG_65	gntP	gluconate permease
	OG_2123	gntR	transcriptional regulator, GntR family
	OG_2441	kdgK*	2-keto-3-deoxygluconate kinase (EC 2.7.1.45)
	OG_3213	-*	4-hydroxy-2-oxoglutarate aldolase (EC 4.1.3.16)
	OG_3214	-	pyridoxal phosphate-dependent enzyme
	OG_3215	-*	amidohydrolase
**gluconate-IV**	OG_737	Pgl	6-phosphogluconolactonase (EC 3.1.1.31)
	OG_45	gntK	gluconokinase (EC 2.7.1.12)
	OG_65	gntP	gluconate permease
	OG_3012	Tal	transaldolase (EC 2.2.1.2)
	OG_2052	Gnd	6-phosphogluconate dehydrogenase, decarboxylating (EC 1.1.1.44)
	OG_58	Tkt	transketolase (EC 2.2.1.1)
	OG_1901	Zwf	glucose-6-phosphate 1-dehydrogenase (EC 1.1.1.49)
**glycerol-3-phosphate**	OG_511	glpT	glycerol-3-phosphate permease
