# Supplementary material for: Linking Bacillus cereus Genotypes and Carbohydrate Utilization Capacity
Source: PLoS One. 2016 Jun 7;11(6):e0156796. doi: 10.1371/journal.pone.0156796 (PMC4896439; doi:10.1371/journal.pone.0156796)
Supplement: S4 Table — (PDF) [file pone.0156796.s004.pdf]

S4 Table. *B. cereus* lactose cassette homology to other species and strains.

	% amino acid sequence identity									Species	Strains
	Glk	LacG	LacE	LacF	LacR	LacA	LacB	LacC	LacD		
Bacillus cereus	99-100	99-100	99-100	99-100	99-100	99-100	99-100	99-100	99-100	cereus	m1293, VD102, VD140, NVH 0075-95, MHI 86
Bacillus sp	-59	64-66			-52	-64	-74	-67	-78	various	
Carnobacterium		65		55-68	-62		-70	-57	-77	various	
Listeria sp		67-69							-69	various	
Streptococcus pneumoniae		64				61				pneumoniae	SMRU2224
Streptococcus agalactiae			63-64							agalactiae	
Streptococcus		-62	63-64	53-59	-50	-63		-56	-76	pyogenes, suis, sanguinis, urinalis, etc	
Clostridium		66	66-70	58	-57	-61	-71	-55		various	
Staphylococcus			62-63	54-61		-62	-70			various	
Lactococcus garvieae			63							garviae	
Lactococcus lactis	-44	63	60	55	44	-64	-68	-53	-59	lactis	various
Enterococcus		-64		53-69	-55		-72	-53	-70	various	
Leptotrichia				59-63				-55		various	
Tetragenococcus						-60					
